# Supplementary material for: Machine learning methods for predicting guide RNA effects in CRISPR epigenome editing experiments
Source: bioRxiv. 2024 Apr 19:2024.04.18.590188. Preprint. [Version 1] doi: 10.1101/2024.04.18.590188 (PMC11042384; doi:10.1101/2024.04.18.590188)
Supplement: Supplement 1 [file media-1.pdf]

Supplementary Files

Wancen Mu et al.

Supplementary Text

Description of annotation features:

Our methodology has been fortified by the careful selection of top annotation features guided by their SHAP values derived from cell fitness prediction tasks in enhancer and promoter regions. From a comprehensive list of annotation features given below, we prioritized six annotations based on their prominence and relevance in the studied context. These annotations, namely H3K4me3, ATAC-seq, H3K27ac, Deltagh, Deltagb, and OGEE_prop_Essential, were chosen for their significant roles and contributions to the gene regulatory mechanisms and cellular functionalities.

H3K4me3: H3K4me3 CPM per 1kb for each DHS. If a DHS entirely overlaps two (or more) tiled genomic regions with 1kb width, the summation is shown.

ATAC-seq: ATAC-seq CPM per 1kb for each DHS. If a DHS entirely overlaps two (or more) tiled genomic regions with 1kb width, the summation is shown.

H3K27ac: H3K27ac CPM per 1kb for each DHS. If a DHS entirely overlaps two (or more) tiled genomic regions with 1kb width, the summation is shown.

TF_GATA2: GATA2 CPM per 1kb for each DHS. If a DHS entirely overlaps two (or more) tiled genomic regions with 1kb width, the summation is shown.

TF_TAL1: TAL1 CPM per 1kb for each DHS If a DHS entirely overlaps two (or more) tiled genomic regions with 1kb width, the summation is shown.

TF_MYC: MYC CPM per 1kb for each DHS. If a DHS entirely overlaps two (or more) tiled genomic regions with 1kb width, the summation is shown.

Deltagb: The free energy metric that quantifies binding affinity of the Cas9–gRNA–DNA complex according to Alkan et al. 2018[29]

Deltagh: The free energy contribution of gRNA–DNA hybridization according to Alkan et al. 2018[29]

olap_number: The number of overlapping Hi-C (High-throughput chromosome conformation capture) pairs[30] with the 20 bp gRNA target site for each gRNA

sum_log10fdr: The total log10-transformed FDR-corrected p-value of the overlapping Hi-C pairs with the 20 bp gRNA target site for each gRNA

prom_olap_number: The number of overlapping Hi-C pairs, where one side is located in the promoter region and the other side is within the 20 bp gRNA target site. The promoter regions of genes were obtained from the gencode.v28lift37.txt.
prom_sum_log10fdr: The total log10-transformed FDR-corrected p-value of the overlapping Hi-C pairs, where one side is located in the promoter region and the other side is within the 20 bp gRNA target site for each gRNA. The promoter regions of genes were obtained from the gencode.v28lift37.txt.
ploidyZhou: Large scale ploidy of the region according to Zhou et al. 2019 (NA value if ploidy of the region was not reported or if gRNA overlap two regions with different ploidy)
LossHetZhou: True if region lost heterozygosity according to Zhou et al. 2019, False otherwise
SV_Zhou: True if structural variant overlap gRNA according to Zhou et al. 2019, False otherwise
SNV_Zhou: True if single nucleotide variant overlap gRNA according to Zhou et al. 2019, False otherwise
conserved_score: select the max score if gRNA overlaps two or more conserved regions according to Zoonomia Project[31].
Conserved: True if gRNA overlap conserved regions (>top90% percentile of 10bp regions) according to di Iulio et al. Nat Gen 2018. False otherwise
Gquad_n_overlap_same_strand: Number of bases of the gRNA overlapping a G4 quad on the same strand (0 if no overlap between gRNA and G4 quad)
Gquad_n_overlap_other_strand: Number of bases of the gRNA overlapping a G4 quad on the other strand (0 if no overlap between gRNA and G4 quad)
distance: distance of the gRNA to the closest protein coding gene on the same strand (gene definition from gencode.v29lift37). Negative if before TSS, positive if after the TES, 0 if within gene boundaries.
medianRNAseqTPM: Median of the mean TPM of 4 ENCODE K562 RNA-seq experiments. The 4 experiments are "ENCSR000AEM", "ENCSR000AEO", "ENCSR000CPH", "ENCSR545DKY"), each with 2 technical replicates. We first took the mean TPM across replicates for each experiment, then took the median of the mean TPMs across the 4 experiments for genes measured in all 4)
probIntolerantLoF: probability that the gene is intolerant to loss of function mutation (from: Lek et al. Nature, 2016)
numTKOHits_Hart: number of cell lines in which the gene is essential (from Hart et al., Cell 2018)

HartEssential: True if genes is essential in more than 2 of the Hart et al. cell lines (their definition of essential genes, genes with 1 or 2 could be defined as conditionally essential)
OGEE_n_Essential: number of cell lines in which the gene is essential according to the OGEE database (http://ogee.medgenius.info)
OGEE_n_NonEssential: number of cell lines in which the gene is non-essential according to the OGEE database (http://ogee.medgenius.info)
OGEE_n: number of cell lines in which the gene was tested for essentiality according to the OGEE database (http://ogee.medgenius.info)
OGEE_prop_Essential: proportion of cell lines in which the gene is essential according to the OGEE database (http://ogee.medgenius.info)
OGEE_prop_NonEssential: proportion of cell lines in which the gene is non-essential according to the OGEE database (http://ogee.medgenius.info)
cancer_census_tier: cancer tier (https://cancer.sanger.ac.uk/). Tier1: To be classified into Tier 1, a gene must possess a documented activity relevant to cancer, Tier2: genes with strong indications of a role in cancer but with less extensive available evidence. Value set to 0 for non-cancer genes.
cancer_census_tissue_type: L: leukemia/lymphoma, E: epithelial, M: mesenchymal, O: other.
sum normalised Hi-C: sum of normalised Hi-C for each DHS (from ABC file). Values are only shown for gRNA entirely within the DHS. If a gRNA entirely overlaps two extended DHS, the mean is shown.

Supplementary Figure

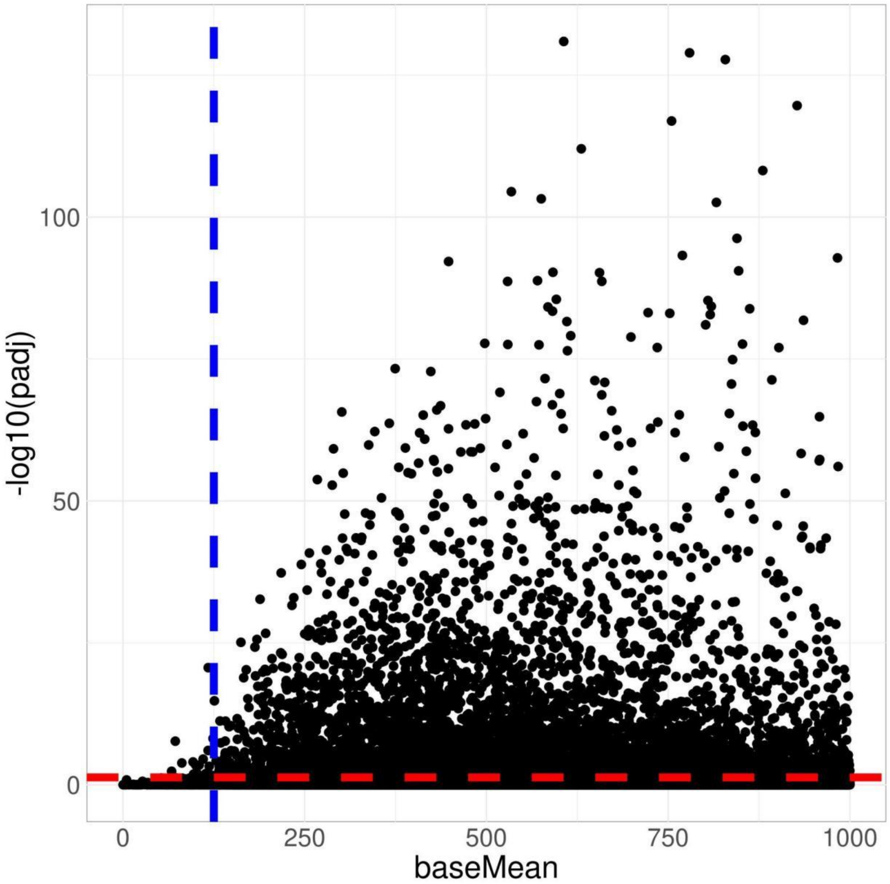

Supplementary Fig. 1 | *Scatter plot of total counts versus -log10(adj.p-value) for all gRNA in wgCERES screen. The blue dashed line represents the threshold of total counts at 125, while the red dashed line represents the threshold of -log10(adj.p-value) at 0.05.*

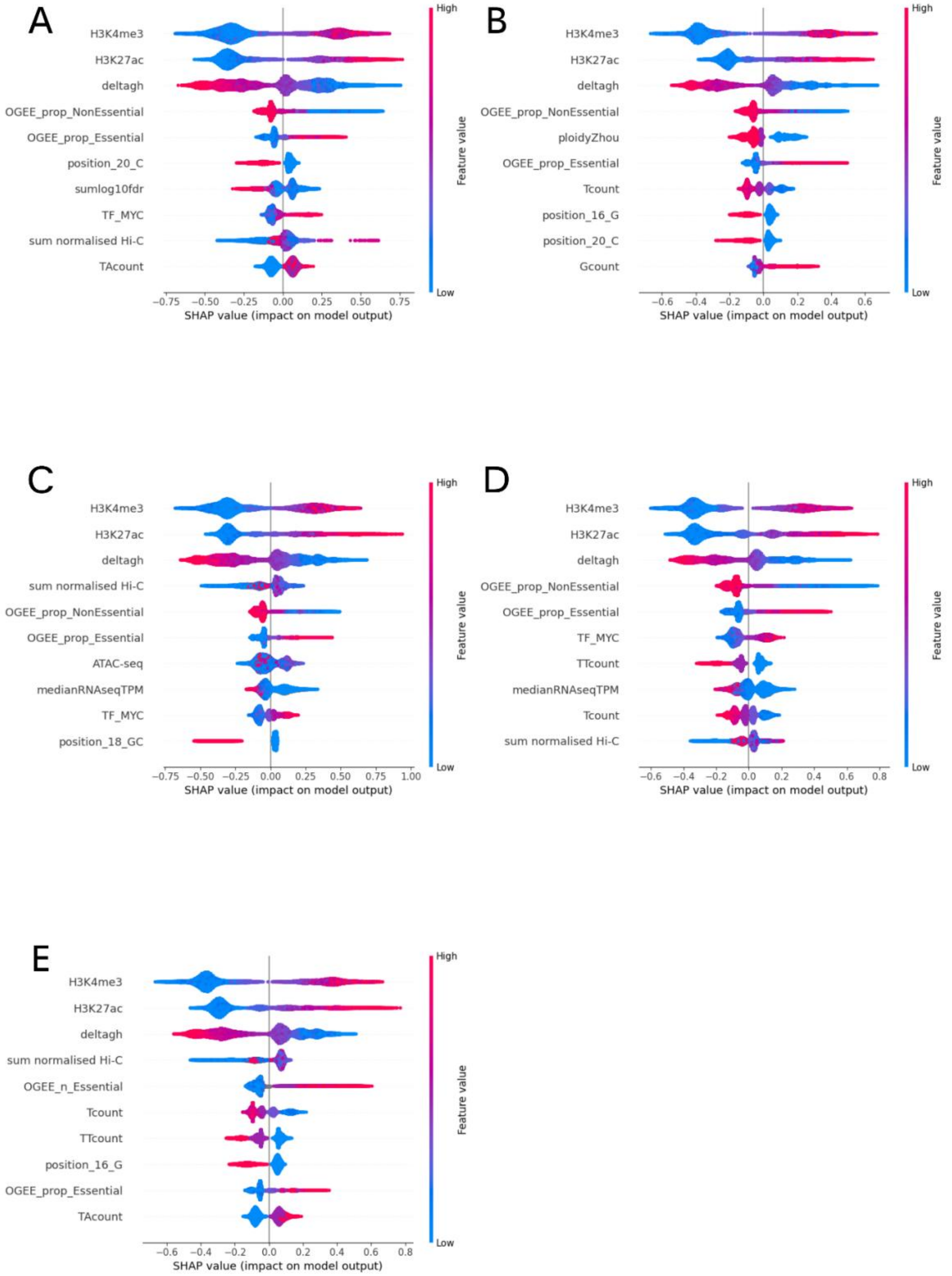

Supplementary Fig. 2 | SHAP summary plot for gRNAs targeting promoter regions. *Top 10 significant features in the XGBoost predictive model for cell fitness, presented separately for each of the 5 folds of gRNAs.*

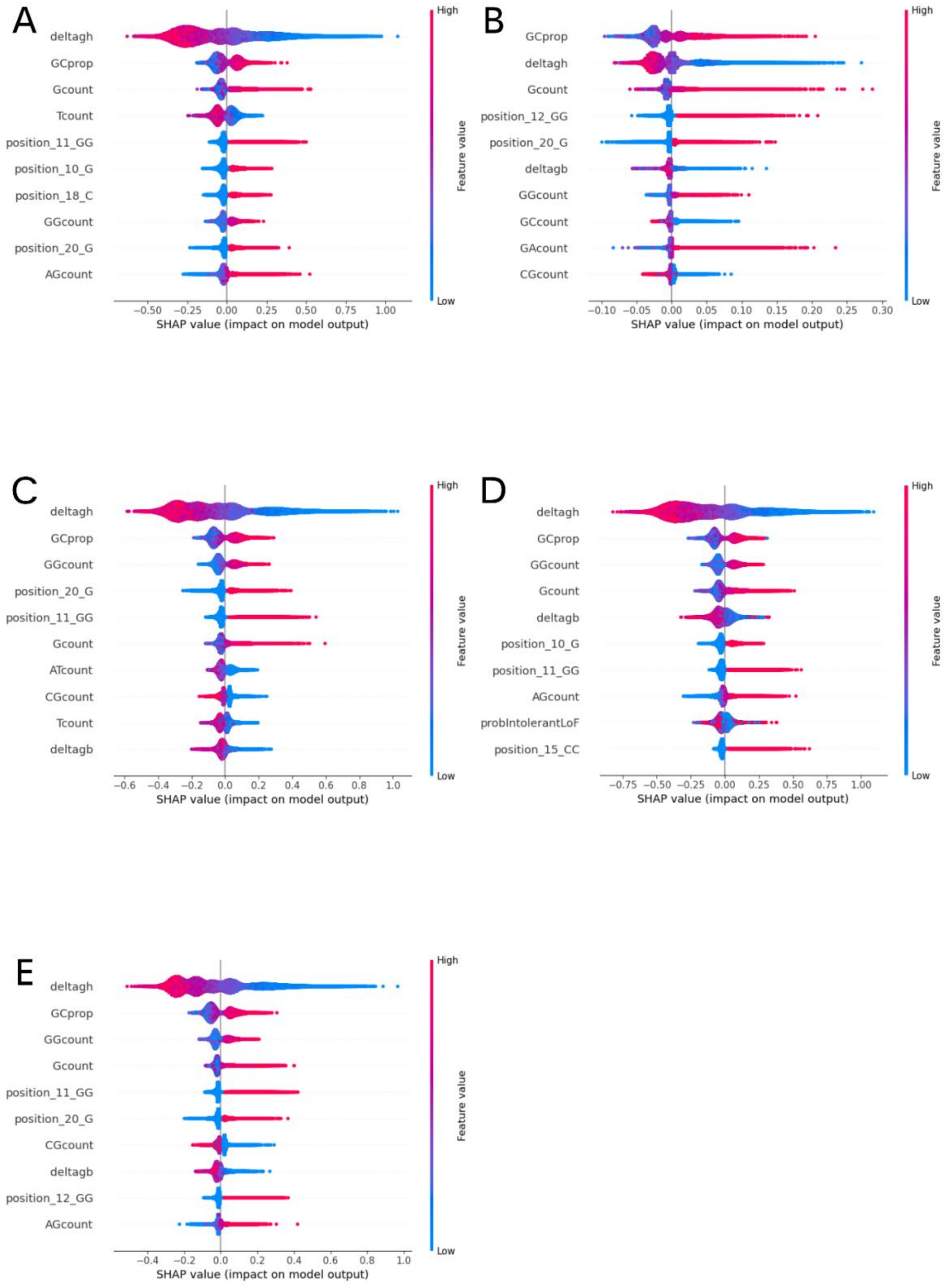

Supplementary Fig. 3 | SHAP summary plot for gRNAs targeting enhancer regions. *Top 10 significant features in the XGBoost predictive model for cell fitness, presented separately for each of the 5 folds of gRNAs.*

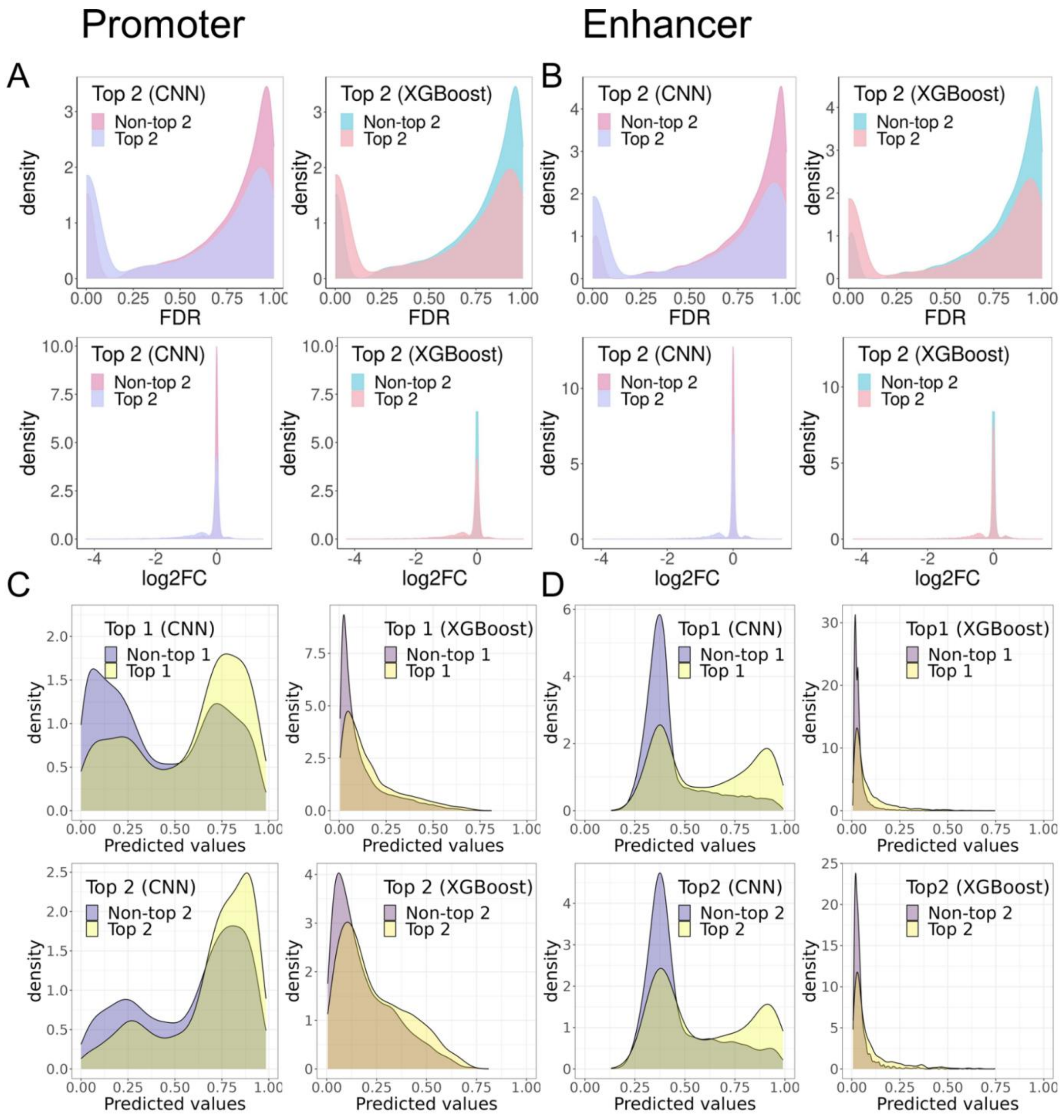

Supplementary Fig. 4 |Predictive performance for cell fitness. *(A,B) Density plots of FDR and log_2FC between the top 2 gRNA selected by CNN and XGBoost and the remaining gRNAs for promoter and enhancer, respectively. (C,D) Density plots of predicted probability of significance by CNN and XGBoost for the top-ranked observed gRNAs and remaining gRNAs in the promoter and enhancer, respectively.*

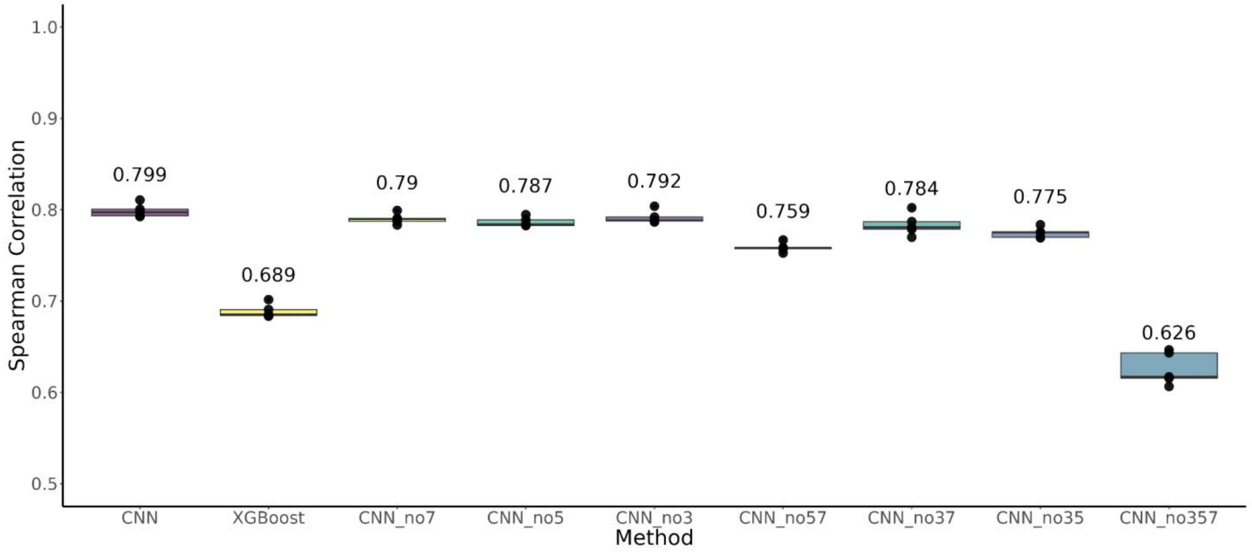

Supplementary Fig. 5 | Prediction performance for counts in wild-type cells. *Mean Spearman correlation across five data folds are listed on top of each box. CNN_no57: CNN without kernels of size 5 and 7 nt; CNN_no357: CNN without kernels of size 3, 5, and 7 nt. P-values from paired t-tests are listed above the bars.*

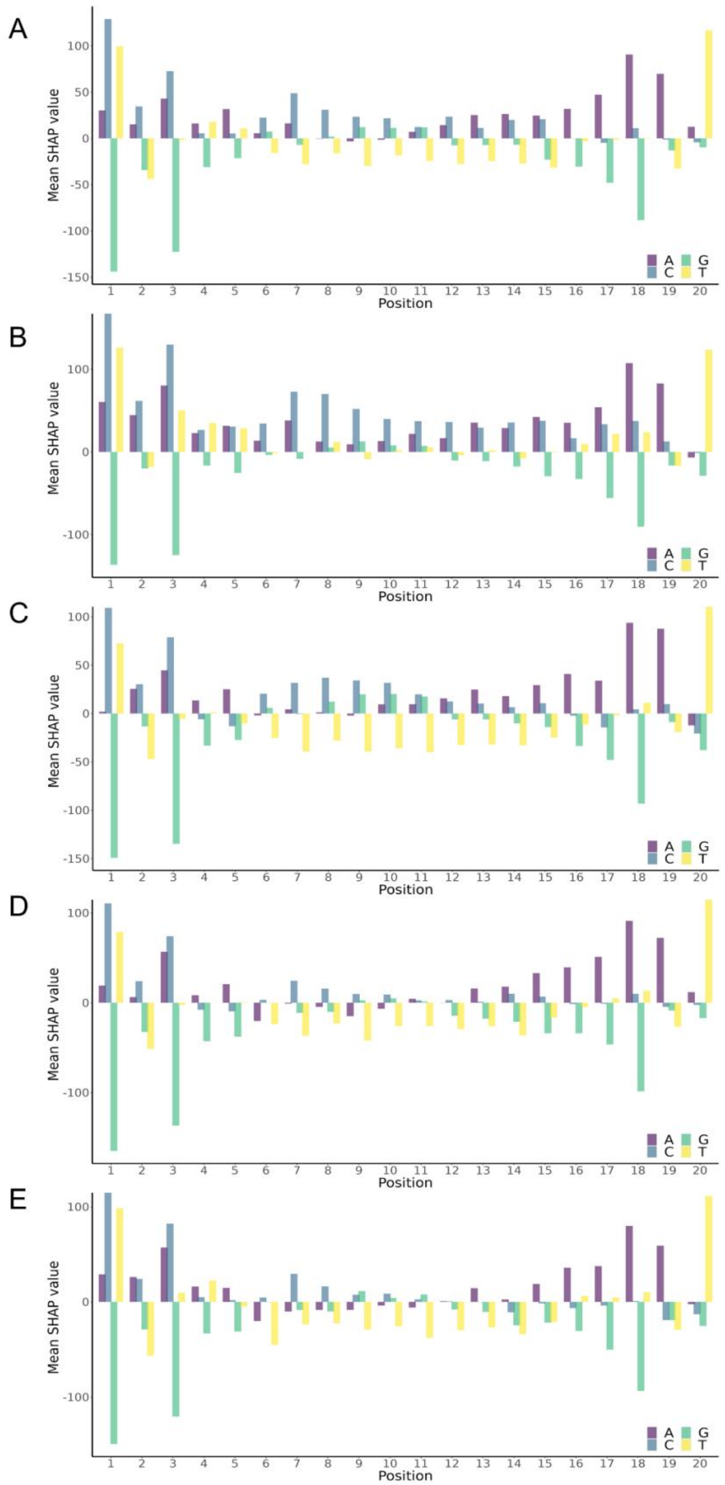

Supplementary Fig. 6 | DeepSHAP summary plot. *Mean DeepSHAP values for input sequence positions in all 5 folds of gRNAs from trained wild-type count CNN models.*

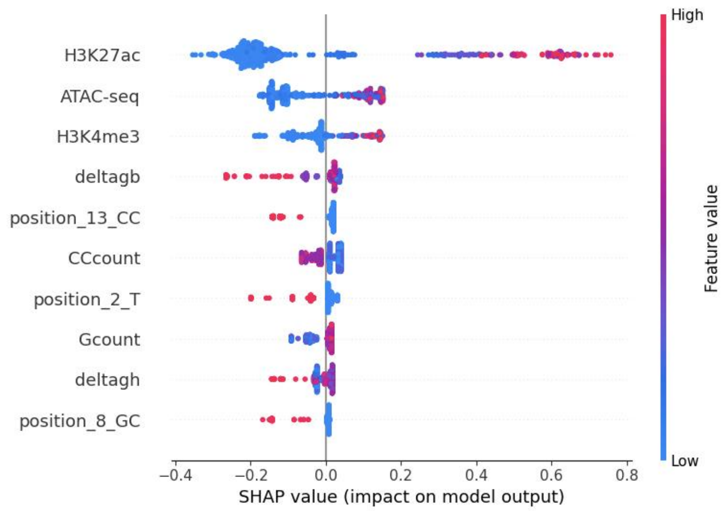

Supplementary Fig. 7 | SHAP summary plot for scCERES. *SHAP summary plot that displays the top 10 significant features for XGBoost across all 5 folds of gRNAs.*

Supplementary Table

Supplementary Table 1A: Sample size in different training folds for cell fitness data

Task	Fold	Chromosomes Included in Test Sets	Significant gRNAs	Insignificant gRNAs
Cell Fitness - Promoter Regions	1	4, 10, 16, 17, 21, X	1,042	62,251
	2	1, 14, 22	775	47,273
	3	2, 7, 11, 13	864	50,450
	4	3, 5, 6, 18, 19	977	64,416
	5	8, 9, 12, 15, 20	815	51,217

Cell Fitness - Enhancer Regions	1	4, 10, 16, 17, 21, X	1,079	157,864
	2	1, 14, 22	853	122,124
	3	2, 7, 11, 13	984	143,593
	4	3, 5, 6, 18, 19	1,030	161,938
	5	8, 9, 12, 15, 20	899	143,187

Supplementary Table 1B: Sample size in different training folds for wild-type cell count data

Task	Fold	Chromosomes Included in Test Sets	Number of gRNAs
Wild-type average counts	1	16, 17, X	1,968
	2	1, 14, 21, 22	1,975
	3	2, 4, 7, 11, 13	1,973
	4	3, 5, 6, 10, 18, 19	2,008
	5	8, 9, 12, 15, 20	1,951

Supplementary Table 1C: Sample size in different training folds for single cell genome-wide screen

Task	Fold	Chromosomes Included in Test Sets	Significant gRNAs	Insignificant gRNAs

Single cell genome-wide screen for K562 cells	1	5, 6, 12, 15, 21, 22	107	450
	2	2, 4, 10, 16, 18	98	403
	3	1, 7, X	104	437
	4	3, 8, 9, 14, 20	77	418
	5	11, 13, 17, 19	126	377

Supplementary Table 1D: Sample size in different training folds for single cell MHC region

Task	Fold	Significant gRNAs	Insignificant gRNAs
Single cell screen in MHC region for K562 cells	1	334	1,562
	2	308	1,599
	3	318	1,634
	4	380	1,567
	5	329	1,519
Single cell screen in MHC region for NPC	1	314	1,550
	2	252	1,618
	3	345	1,544
	4	387	1,531
	5	294	1,501

Single cell screen in MHC region for iPSC	1	519	1,374
	2	530	1,397
	3	513	1,364
	4	578	1,358
	5	524	1,413

Supplementary Table 2A: Comparing gRNAs impact on cell fitness vs gRNAs impact in at least one gene expression

	gRNA significant in at least one gene expression	gRNA insignificant in any gene expression
gRNA significant in cell fitness	295	586
gRNA insignificant in cell fitness	144	413

Supplementary Table 2B: Comparing gRNAs impact on cell fitness vs gRNAs impact in larger than three gene expression

	gRNA significant in larger than three gene expression	gRNA insignificant in any gene expression
gRNA significant in cell fitness	21	860
gRNA insignificant in cell fitness	2	555

Supplementary Table 3: ENCODE human functional annotations

File name	Biological replicate	Type
**K562**		
ENCFF512VEZ.bam	Rep1	ATAC-seq
ENCFF987XOV.bam	Rep2	ATAC-seq
ENCFF656DMV.bam	Rep1	H3K4me3
ENCFF440ARP.bam	Rep2	H3K4me3
ENCFF907MNY.bam	Rep1	H3K27ac
ENCFF907MNY.bam	Rep2	H3K27ac
ENCFF307XGY.bam	Rep1	GATA2
ENCFF032UMK.bam	Rep2	GATA2
ENCFF304UTB.bam	Rep1	TAL1
ENCFF631RZK.bam	Rep2	TAL1
ENCFF503ZCR.bam	Rep1	MYC
ENCFF604CDP.bam	Rep2	MYC
**WTC11 iPSC**		
ENCFF235SBC.bam	Rep1	ATAC-seq
ENCFF735XZN.bam	Rep2	ATAC-seq
ENCFF979XKI.bam	Rep1	H3K4me3
ENCFF479NLV.bam	Rep2	H3K4me3
ENCFF738QRT.bam	Rep1	H3K27ac
ENCFF696GDY.bam	Rep2	H3K27ac
**NPC**		
ENCFF589KAY.bam	Rep1	H3K4me3
ENCFF212FUS.bam	Rep2	H3K4me3
ENCFF805URT.bam	Rep1	H3K27ac

ENCFF120AVO.bam	Rep2	H3K27ac
ENCFF477FHN.bam	Rep3	H3K27ac
